# What is day hospital treatment for anorexia nervosa really like? A reflexive thematic analysis of feedback from young people

**DOI:** 10.1186/s40337-023-00949-y

**Published:** 2023-12-14

**Authors:** Lucinda J. Gledhill, Danielle MacInnes, Sze Chi Chan, Charlotte Drewery, Charlotte Watson, Julian Baudinet

**Affiliations:** 1 South London and Maudsley NHS Foundation Trust, UK South London and Maudsley NHS Foundation Trust, Bethlem Royal Hospital, Monks Orchard Road, Beckenham, Kent, BR3 3BX UK; 2 Department of Psychology, Institute of Psychiatry, Psychology and Neuroscience, King’s College London, 16 De Crespigny Park, London, SE5 8AF UK; 3 Present Address: Intensive Treatment Programme of the Maudsley Centre for Child and Adolescent Eating Disorders, Michael Rutter Centre, Maudsley Hospital, Denmark Hill, London, SE5 8AZ UK

**Keywords:** Anorexia nervosa, Adolescents, Day programme, Experience, Feedback

## Abstract

**Background:**

A significant proportion of young people do not respond to the NICE recommended treatment for anorexia nervosa: Family Therapy. Whilst historically these young people would be admitted to inpatient services, which are associated with greater treatment cost, greater risk of relapse, and worse outcome, more recently evidence is building for the effectiveness of day programmes. One day programme that has been found to be effective is the Intensive Treatment Programme (ITP) of the Maudsley Centre for Child & Adolescent Eating Disorders in London, UK. However, to-date no studies have investigated how young people experience such a day programme.

**Method:**

Anonymous feedback was completed via online survey by 51 young people over a 5-year period (2018–2023) on discharge from ITP.

**Results:**

Four main themes were identified: (1) Support—young people expressed the importance of boundaries but also of feeling validated, and encouraged; (2) Uniqueness: an experience like no other—ITP was described as different to any other treatment received before (both outpatient and inpatient); (3) Relationships – young people valued connecting with others in a similar situation and reflected that relationships at home changed throughout treatment; (4) Self-development – learning skills, developing independence, and exploring an identity outside of the eating disorder was valued.

**Conclusions:**

It is hoped that the reflections from these young people can help to inform clinicians working in DPs and those hoping to set up novel DPs about key aspects of treatment.

## Abstract (plain language summary)

This study examined the experiences of young people who attended an intensive treatment day programme (ITP) over a five-year period. 51 young people completed online questionnaires up to one-month after being discharged from the programme between 2018 and 2023. All young people were between the ages of 11–18 and had a diagnosis of anorexia nervosa. The authors explored all free text responses from the young people (for example: what were the most important parts of ITP) and found four common themes. These were: (1) Support – feeling validated, encouraged, and finding boundaries helpful, (2) Uniqueness: an experience like no other – treatment was different to any received before, (3) Relationships – connecting with others in a similar situation and relationships at home changing, (4) Self-development – learning skills, finding their identity, and developing independence. It is hoped that the reflections from these young people can help to inform clinicians working in DPs and those hoping to set up novel DPs about key aspects of treatment.

Family therapy for anorexia nervosa (FT-AN) [1] is the recommended first line treatment for children and adolescents in the UK [2]. Nevertheless, there is still a significant proportion of young people for whom FT-AN is not effective [3–5] with up to 50% not reaching full remission by the end of treatment [6].

Historically, young people who have not responded to treatment in community settings have required inpatient treatment, which is associated with very high treatment costs and an increased rate of relapse and re-admission [7]. In recent years, brief outpatient intensive treatment options (3–6 months), such as day programmes (DPs), are beginning to be developed. A recent systematic scoping review highlighted that DPs are an effective model for supporting physical and psychological changes for very unwell young people while they remain in the community [8]. One randomized controlled trial (RCT) demonstrated that DP treatment after a brief inpatient admission was no less effective than longer inpatient admissions in regard to weight restoration and maintenance for a year post-admission [9].

The Intensive Treatment Programme (ITP) is a day programme for adolescents (aged 11–18) with restrictive eating disorders. It operates five days per week and is based at the Maudsley Hospital in London, UK. Outcomes suggest ITP is effective in supporting improvements in both physical health and psycho-social factors such as eating disorder psychopathology, depression and anxiety, quality of life, relationship and attachment quality, and emotional overcontrol [10, 11]. Qualitative data on the perceived change mechanisms within an ITP suggest the following as important: (1) the intensity and structure of the programme, (2) the connection with other staff, young people and families, and (3) the focus on generalising new learning beyond ITP. These three key aspects together are thought to instil hope, increase mentalization, and ultimately facilitate change [12].

However, despite the increasingly widespread use of day programmes internationally, little is known about the young person’s experience of being in an intensive day programme. The only available data suggests young people prefer it face-to-face, compared to virtual delivery, as was trialled during the COVID-19 pandemic [13]. Over a 5-year period, anonymous qualitative feedback was collected at discharge from young people who had been through ITP.

## Methods

### Design

A convenience sample of young people’s perception and experience of treatment was explored through the use of anonymous feedback questionnaires. All young people who completed treatment in ITP at the Maudsley Centre for Child and Adolescent Eating Disorders (MCCAED) between May 2018 and March 2023 were invited to give feedback about their experience, regardless of treatment outcome. Feedback questionnaires were intentionally anonymous as this was thought to encourage openness and reduce the possibility of demand characteristics.

## Treatment setting

ITP operates five days per week (Monday to Friday) in south London, UK. It is one part of MCCAED, a large, publicly funded, specialist child and adolescent eating disorder service. All young people who attend the programme receive weekly family and individual therapy, daily therapeutic groups, three to four meals supervised per day with staff, and education support. The therapeutic group programme is predominantly based in radically open dialectical behaviour therapy adapted for adolescents (RO DBT), cognitive behavioural therapy (CBT), cognitive remediation therapy (CRT) and art therapy. It is a rolling open-ended programme with a mean length of stay approximately 13 weeks. See previous outcome studies for further description of the programme and treatment response [10, 11].

## Recruitment

Feedback questionnaires were sent out to all young people who completed the programme via email within 1 month of discharge. All feedback was anonymous, and no forced responses were used, meaning young people could answer as many or as few of the questions as they chose. Between March 2020 and November 2020, the programme changed significantly due to restrictions on face-to-face working during the coronavirus (COVID-19) pandemic. Feedback from this period was not included in this study given the programme was so different to how it typically operates. Young person and parent experiences of online ITP during this period are reported elsewhere [13].

## Data analysis

The feedback was analysed using Reflexive Thematic Analysis [14]. The data were approached within a critical realist framework. Experience and meaning are considered subjective and influenced by social and cultural context. An inductive and semantic approach was used to analyse the data. The two analysing authors (DM and SZC) were guided and directed by the explicit content of the data rather than making assumptions, making inferences or looking at existing concepts [15, 16].

Thematic analysis, as outlined in six phases by Braun and Clarke [16], was conducted. Two authors separately and independently immersed themselves in the data. Following this, analysing authors generated codes and themes (again independently) and then met to discuss. Several one-hour meetings were held to discuss the process, with time in between each meeting to allow for themes to be revised before coming to a final agreement. As noted by Braun and Clarke [16], the process of generating themes is ‘creative and active’, therefore theme frequency was not noted.

The two analysing authors, DM and SZC, were new to ITP and therefore had no prior conceptions about the effectiveness of the programme or young people’s experience of treatment. It was felt that this provided an extra level of objectivity to the thematic analysis. The analysis was overseen by author JB and CW, who met with DM and SZC on three separate occasions during the analysis process. Themes were finalised via an inductive approach.

## Results

### Sample

124 adolescents attended ITP between May 2018 and March 2023. Of these, 28 attended ITP during the period of significant changes due to COVID-19 restrictions and incomparable to standard operating before and after this time period. Their feedback is not included in this study.

51 of 96 young people (53.13%) completed written feedback about their experience of treatment. All young people had a DSM-5 [17] diagnosis of a Anorexia Nervosa or related restrictive eating disorder (Anorexia Nervosa – restrictive subtype or binge purge subtype, or Atypical Anorexia), and were between the ages of 11–18 years old.

## Qualitative findings

Four themes and eight subthemes were identified in the analysis (see Table 1).

**Table 1:** Themes and subthemes

Main themes	Sub-themes
1. Support	1a. Feeling validated and accepted
	1b. Rules, Structure and Reliability
	1c. Pushing and Encouragement
2. Uniqueness: an experience like no other	
3. Relationships	3a. Within ITP
	3b. Outside ITP
4. Self-Development	4a. Psychological Skills
	4b. Rediscovering Self
	4c. Returning to Normality

## Theme 1: support

ITP was frequently described in terms of providing a framework of ‘intensive support’. This was experienced by young people through multiple levels/ mechanisms. Intensive support was noted as being a key factor in young people reaching their goals, transitioning onwards from ITP back to the community and whether they would recommend ITP to another young person experiencing similar challenges. Support was experienced through:

1. Feeling validated and accepted

Young people frequently expressed feeling validated and accepted by the team and how this was a supportive factor in their journey towards recovery. This was expressed in various ways, for example, through ‘being listened to’, by ‘being involved’, ‘given choices’ and having a ‘say’ in their treatment. Others felt a sense of ‘acknowledgement and understanding’ from the team.

*“My team listened to what I was saying”* and “*I felt listened to… [and understood]”.*

*“Involved me in every step of treatment and gave me a voice and a say in what happens”.*

*“All of the team listen to you and take your views into consideration with your plan so you feel like you are understood and acknowledged’.*

Conversely, some young people did not feel validated or listened to. This was experienced negatively and as a barrier to being able to fully engage with the programme as well as move towards recovery.

*“I felt like I often wasn’t being listened to or would get dismissed because my ‘anorexic brain couldn’t think properly’ which is true to an extent but I also felt like my opinions weren’t heard”*

*“I didn’t really have any say, which I think would be better if that changed”.*

*“Having to eat things that I did not like was hard and unhelpful as it made me feel that no one listened to me”.*

1. Provision of Rules, Structure and Reliability

The supportive nature of ITP was frequently reflected on in terms of the ‘rules, structure and reliability’ that ITP provides. This was reported in both a general sense and also specifically in relation to goal setting, being regularly encouraged and through the strict nature of the programme.

*“Regular and reliable individual sessions…”*

“*Having…[meal replacements] and strict rules”*

‘Goal setting’ was described as being a positive and helpful aspect of the programme and the ‘strictness’ of adhering to the rules was also reported as being beneficial.

*“…making me choose three goals at the beginning*”

*“…so everyone can meet their goals”*

For some young people, the intensive and strict environment was spoken of in more negative terms. This was described in relation to both the idea that ITP was ‘too strict’, while for others it wasn’t ‘strict enough’.

*“It would be nicer if they were less strict though”*

*“More rules about interaction between young people / control on triggering behaviours”*

1. Pushing and Encouragement

Many young people reported the feeling of being ‘pushed’ and encouraged to meet their goals and to stay on track. This was generally described in positive terms and was seen as contributing to a successful outcome. Some young people also specifically noted the significance of the presence of encouragement ‘despite difficulty’. This was in the context of persevering even though it was hard and reinforces the intensive, reliable and structured nature of ITP.

*“It helped me start my recovery and pushed me further than ever”*

*“Having people to support me even when things were difficult”*

## Theme 2: uniqueness: an experience like no other

Young people described ITP as a unique experience that was distinct from other treatments they have previously experienced. They generally felt that ITP was a better option and recommended ITP over other treatment modalities. This is because they felt that ITP allowed young people to prevent institutionalisation/ hospitalisation and achieve recovery whilst still living a normal life (i.e. attending mainstream education, staying at home).

*“…have been in multiple different types of therapy, units... and nothing has helped like ITP has”*

*“…the way it [ITP] integrates life at home and ITP has proven far more useful and effective than inpatient units”*

Specifically, an important comparison young people made between inpatient admission and ITP was the continuity of their recovery. One young person noted that the progress made during ITP was much better maintained upon discharge compared to inpatient treatment. One young person suggested that this is because ITP provides a more suitable recovery plan. Others suggested that ITP addressed eating at home, tackles individuals’ difficulties, and offers help to parents, essentially enabling a smooth transition between ITP and home.

*“I am more able to challenge foods at home now, compared to inpatient, where home eating was never really addressed”*

Although, there were some disparities as some young people mentioned that it was difficult to maintain the progress they made at home and would like to have further support.

*“Helped in terms of actually normalising eating again, but it was very hard to keep that going with continuity at home and sometimes the fact it was almost impossible to enforce at home meant that the progress made in ITP was undermined”.*

## Theme 3: relationships as a vehicle to recovery

References to relationships were frequently mentioned throughout the comments. They were described in both positive and negative terms and can be categorised as those relationships existing within ITP as being distinct to those outside of ITP.

- Relationships within ITP - Peer and Therapeutic

Within ITP, comments related mainly to peer/social relationships and therapeutic relationships with staff. Young people reported a general increase in sociability with peers within the group which was seen in positive terms. This resulted from learning how to better deal with social situations, generally feeling ‘brighter’ and through being in an environment that allowed the formation of friendships with the other young people on the programme.

*“Friendships with the other young people”*

*“I found it really helpful to be there with other girls and talk about things other than eating”*

Relationships within ITP were also expressed in positive terms in relation to experiencing a sense of ‘belonging, camaraderie and being part of a ‘tribe’. Young people reported feeling a ‘shared bond’ with others on the programme which contributed positively to a collective sense of ‘I am not alone’.

*“Community within the tribe”*

*“…so we were all in the same boat”*

*“Being around the other YP who were struggling just as much as me and seeing they could eat made me believe I could too”*

The feeling of a shared experience was reported not just by the individuals themselves but also referenced in relation to their parents and families.

*“It really helped my parents cope and meet with other families going through the same things”*

3b. Relationships outside ITP - Family & Social

Young people frequently reported that their family relationships had benefited from taking part in the programme. This supported young people to move towards recovery by nurturing a more ‘honest, open, trusting and understanding’’ atmosphere at home. They also experienced less negativity towards their parents and an easing of tension within the family. Similarly, several young people found that sibling relationships also improved as a consequence of the programme.

*“Things are so much easier and happier and the tensions has been massively eased”*

*“Our [parental] relationship feels less hindered by the disorder”*

*“Now that things are a bit better between us all, I feel like I can talk more openly with brother”*

In addition, social/ peer relationships outside of ITP were also improved. This was based on a perceived increase in social skills, confidence in handling social situations and experiencing a more upbeat mood which was attributed to being able to eat more and thus have more energy and motivation to socialise.

*“Me eating more made me a lot more bright and social, therefore making my relationships a lot better”*

*“It’s helped me socially”*

*“...a positive impact on friendships”*

*“It [ITP] taught me how to deal with social situations”*

Conversely, some young people reported that relationships had suffered and become more strained due to their participation in ITP. This was reported in the context of a loss of trust and an increase of strain, tension and arguments among family members.

*“I felt that my relationship with my family has been placed under great strain during my time in ITP”*

*“Made me even further away from my brother and they tried to make more work on my relationship with my Dad but it didn’t work…”*

*“ITP made it worse as I lost trust in my mum and argued with her”*

*“Created more tension especially in family sessions”*

## Theme 4: self development

Young people reported an experience of self-development during their time at ITP. This occurred as young people learned psychological skills to cope with their challenges which helped them rediscover themselves and prepare them for life after ITP.

4a Psychological Skills

Young people frequently reported learning a variety of skills that they used to challenge their eating disorder, cope with their anxiety and worries, and tackle perfectionistic traits. Contrarily, others mentioned that there wasn’t enough focus/content on tackling the eating disorder and that they rather avoided the topic during groups. The relatability or applicability of skills was also important to young people. They appreciated that the skills were highly relatable and that they could implement into their everyday life or routines outside of ITP. This included skills on how to deal with overwhelming situations, as well as more practical skills such as knowing what normal eating looks like and how to prepare food for themselves.

*"[ITP] taught me useful skills that I could practice at home and it felt really related to me"*

*“[I] find it easier to prepare food for myself now”.*

4b. Rediscovering Self

Young people expressed that before starting ITP, they felt disconnected from themselves and struggled to find their own identities without the eating disorder. Throughout ITP, young people reported that they are now more aware of their thoughts, feelings, and emotions and essentially learnt more about themselves. This process of self-rediscovery also allowed young people to think about life outside of the disorder.

*“I’ve struggled trying to find my identity without anorexia”*

*“I learnt a lot more about myself and what was fuelling the disorder”*

4c. Onwards and returning to normality

Young people reported gaining independence and autonomy in their lives as a result of recovery. They realised that as they progressed in their recovery, they were able to make more of their own choices and decisions, and were no longer entirely dependent on their parents and/or families. They also described that life was returning to normal, where food is no longer the focus and families could function normally again.

*“…helpful in becoming independent after [ITP] and not relying entirely on parents”*

*“[parents] role wasn't only to look after me, they could also act like parents in a more normal functional way”*

*“…it made me like a normal teenager for once”*

## Discussion

This study aimed to understand the lived experiences of young people who had been through an ITP. Analysis of anonymous qualitative feedback collected over a five-year period identified four main themes “Support”, “Uniqueness: an experience like no other”, “Relationships as a vehicle to recovery”, and “Self-development”.

The intensity of the support received was a key factor– this was experienced both practically through the rules and structure of the programme which provided reliability to the care being given, and psychologically through feeling validated and accepted. The ability of staff to hold boundaries around eating disorder behaviours consistently and repeatedly throughout the programme seemed to be considered a challenging, yet needed element of the programme by the majority of young people.

The idea that ITP provides a unique experience is an interesting one as the foundation of treatment is the same as in outpatient settings–the main treatment model used at ITP is FT-AN [1]. The elements that young people highlighted as making ITP different to previous outpatient treatment (attending mainstream education, staying at home, having elements that specifically address eating at home, group-based psychological treatment targeting psychosocial difficulties related to eating disorders) reflect the ethos the service holds of making life bigger for the young people and the importance of change happening both at ITP but also simultaneously at home.

The theme of ‘Relationships as a vehicle to recovery’ highlights the importance of treatment being broader than just symptom management. It also mirrors the theoretical model of change described in FT-AN [1], and fits with qualitative exploration of perceived change mechanisms within intensive FT-AN informed treatment [12, 18]. Specifically, it suggests that when sufficient progress is not being made, it is important to refocus the therapeutic work on difficulties in relationships within the family. This supports the different sub-themes mentioned between relationships within ITP – peer and therapeutic – and relationships outside ITP – family and social. The relational framework of FT-AN states that the clinician’s self is an important part of the treatment process, and that the strength of the therapeutic relationship can drive change/recovery. If one considers ITP to be a step up from outpatient treatment, it could be expected that there needs to be a step up in the intensity of the therapeutic relationship as part of this.

The theme of self-development through ‘psychological skills’, ‘rediscovering self’ and ‘onwards and returning to normality’ may highlight the role of the different therapeutic groups in ITP. The groups are designed to help young people learn new skills and become more aware of patterns in their thoughts, emotional responses, and resulting behaviours. This may have helped the young people take important steps on the road to recovery, in turn allowing them more space for autonomy and independence and therefore returning to more regular adolescent development.

## Clinical implications

Clinical implications from the themes generated should encourage professionals to consider when exactly a service can be deemed as “intensive” both in terms of the overall format but also how much time clinicians can dedicate to each family. Therapeutic groups may play a role in the theme of self-development, helping young people rediscover themselves without the eating disorder and having the skills to do this. Clinicians need to consider the group dynamics that may occur and nurture cohesiveness and camaraderie.

Similarly to research into other intensive treatments such as family-focused intensive treatment weeks [19] or multi-family therapy [20], ITP allows clinicians to model firm boundaries and provide coaching to parents on a regular basis. Containment and atonement are described as key concepts in MFT in creating a safe holding environment [21–23]. Families are initially contained by the clinician, with both parents and young people becoming gradually less dependent and more autonomous over time. It could be suggested that in these intensive settings (including ITP), clinicians are taking on the role of a temporary surrogate parent at a time when young peoples’ parents/carers are at their limit. Thought needs to be given to the capacity for day programme clinicians to be available for additional ‘pushing and encouragement’ that the young people referenced as being helpful when things felt difficult. Being available to remind young people of their goals throughout the week and helping them to stay on track was seen by most young people as contributing to a successful outcome. Clinicians also need capacity to consider how to navigate the tightrope between being firm and supportive and to receive support themselves to continue to persevere and encourage ‘despite difficulty’. The elements mentioned in this theme raise questions around what might have been getting in the way of young people feeling validated and accepted in their outpatient treatment.

In addition, some young people spoke of valuing ‘being involved’, ‘given choices’ and having a ‘say in their treatment’, which can feel at odds with the first phases of FT-AN treatment. Early in FT-AN (Phase 1 and 2), parents are often required to take a firm lead on feeding their young person and making difficult decisions on their behalf at a time when their eating disorder cognitions are too strong for them to make healthy choices for themselves. Day programme clinicians will therefore need to strike a difficult balance between holding in mind the importance of parents taking an active, and often firm, role at mealtimes, whilst also acknowledging the young person’s voice and giving choice where appropriate. This feedback really highlights the importance of not losing sight of the young person outside of the illness and trying to promote adolescent development wherever possible.

The results of this study closely map on to the findings by Colla, et al. [12], suggesting that the young people’s experiences of an ITP matched what young people and parents consider to be the mechanisms of change within a day programme. It is also worth noting that the young peoples’ responses also correspond to elements related to the different phases of treatment in FT-AN [1] and perceived change mechanisms of multi-family therapy [18]. The theme of Support (feeling validated; rules, structure and reliability; pushing and encouragement) overlaps with the change mechanism of ‘The intensity and structure of the programme’ as well as the aim of FT-AN Phase 1 to create a safe base and Phase 2 to help the family manage the eating disorder. The theme of Relationships (within ITP; outside ITP) links to the change mechanism of ‘The connection with other staff, young people and families’ and the more general focus of FT-AN Phase 1 – Engagement and development of the therapeutic alliance. The theme of Self-Development (psychological skills; rediscovering self; returning to normality) overlaps with the change mechanism of ‘Focus on generalising new learning beyond ITP’ and elements referenced in FT-AN Phase 3 – Exploring issues of individual and family development. Future research would make a valuable contribution by exploring the experiences of parents and how these compare to the different elements of mechanisms of change, the aims of the different phases of FT-AN, as well as the young peoples’ own experiences.

## Limitations

Despite 96 eligible young people receiving treatment from ITP during the five-year timeframe only 53.13% gave feedback. This creates challenges when deciding if the findings reflect a shared lived experience or are only representative of this sample. In order to encourage openness, the feedback responses were anonymous, meaning feedback responses and treatment outcomes cannot be linked. This anonymity results in a lack of context to the responses. Elements that it would be helpful to know include if there were any comorbidities, if there were any neurodiversity present that may have interacted with or presented a barrier to treatment, what was happening in terms clinical recovery and weight restoration.

It is unknown if those young people who reported more challenging experiences in ITP were struggling more clinically or not (i.e. not reliably restoring weight), compared to those who experienced ITP in a more positive way. It could be hypothesised that those with more positive views of ITP experienced earlier weight gain and thus were able to engage more fully with the programme; whereas young people with negative views of the programme who potentially felt unheard may have not experienced early weight gain and therefore struggled to engage with other young people/staff/therapeutic group work. Given that research suggests that early weight gain is a key factor in the success of FT-AN [24], it would be interesting to look at differing experiences of ITP based on weight trajectory through treatment.

Future work would also benefit from information about the experience of young people who have Autism Spectrum Conditions (ASC) or other features of neurodiversity. We know that behavioural and cognitive rigidity can be a feature in both AN and ASC, with research suggesting that those with both diagnoses have more severe presentations, and poorer treatment outcomes [25–27]. Whilst for some young people with ASC following such a strict programme with lots of rules and expectations may be containing, for others it may be more challenging. Knowing this information could potentially lead to important discussions on adaptations to a DP that can be made for young people with neurodiversity, in order to help them engage in such a structured programme whilst also feeling heard and validated [28].

In addition, information about the trauma history of this group of young people is not known. It would have been interesting to know whether any of the young people who struggled with aspects of ITP did so because of a resurgence of trauma symptoms. With this in mind, future work would benefit from including this information – does the increased awareness of thoughts and exposure to uncomfortable feelings highlighted in the feedback pose a barrier to engagement for young people with a trauma history who may seek to numb emotions through restriction? Does the initial focus on weight gain and rigidity around this lead to this group of young people feeling invalidated and misunderstood by professionals?

As above, this lack of demographic information/ symptomatology impacts on future research that could influence clinical practice as it means that we will be unable to link young people’s experiences to their corresponding parent responses. This would help bring further context to the experiences. However, the themes that emerged in this study triangulate with previous research findings, suggesting that they are shared qualities of the experience of young people attending ITP.

## Conclusion

The UK is currently experiencing a cost-of-living crisis, with considerable financial strain placed on public health services. With the high cost of inpatient treatment and uncertain benefits beyond medical stabilisation, it could be hypothesised that there will be an increase in alternative, more cost-effective, intensive services such as day programmes.

This is one of the first qualitative studies to explore young peoples’ experiences of a day programme treatment with a relatively large sample size. Findings highlight the multi-faceted nature of this treatment approach. Expanding our understanding of young peoples’ experiences of day programme treatment is critical in order to continue to improve and refine them, and also to gather new ideas about how to better support those for whom outpatient treatment has not been effective. It is hoped that this study may provide a framework of reference to clinicians working in day programme services to provide meaningful care to the young people and families they have been set up to support.

## Data Availability

Data are available from the corresponding author on reasonable request.

## Abbreviations

COVID-19: Coronavirus Disease 2019
DP: Day Programme
FT-AN: Family Therapy for Anorexia Nervosa
ITP: Intensive Treatment Programme
MCCAED: Maudsley Centre for Child & Adolescent Eating Disorders, Maudsley Hospital, UK
NICE: National Institute for Clinical Excellence
RCT: Randomised Controlled Trial
